# Sexual and reproductive health services (SRHS) for adolescents in Enugu state, Nigeria: a mixed methods approach

**DOI:** 10.1186/s12913-017-2779-x

**Published:** 2018-02-08

**Authors:** Amelia Ngozi ODO, Efiong Sunday SAMUEL, Evelyn N. NWAGU, Petra Obioma NNAMANI, Chiemezie S. ATAMA

**Affiliations:** 1 Department of Human Kinetics and Health Education, University of Nigeria, Nsukka, Enugu, 410001 Nigeria; 2 Department of Pharmaceutics, University of Nigeria, Nsukka, Nigeria; 3 Department of Sociology/Anthropology, University of Nigeria, Nsukka, Nigeria

**Keywords:** Availability, Accessibility, Sexual and reproductive health services, Adolescents, Mixed method

## Abstract

**Background:**

Availability and accessibility of sexual and reproductive health services for adolescents are very crucial for prevention and control of sexual and reproductive health problems. These services also play vital roles in the promotion of adolescents’ sexual and reproductive health generally. The main purpose of the study was to determine the availability and accessibility (geographical and financial) of sexual and reproductive health services (SRHS) among adolescents in Enugu State, Nigeria.

**Methods:**

A mixed methods approach was adopted for the study. 192 health facilities were reached to check availability of SRH services. Randomly sampled 1447 adolescents (12–22 years) completed the questionnaire correctly. Twenty-seven interviews and 18 group discussions were conducted. Instruments for data collection consisted of a checklist, a questionnaire, a focus group discussion guide and an in-depth interview guide. All instruments were pre-tested. Quantitative data were analyzed using descriptive statistics and Chi-square tests. NVivo 11 Pro software was used to code and thematically analyze the qualitative data.

**Results:**

A total of 1447 adolescents (between 12 and 22 years) completed the questionnaire correctly. Among these adolescents, males constituted 42.9% while females were 57.1%. The majority (86.7%) of the adolescents reported availability of safe motherhood services, and 67.5% reported availability of services for prevention and management of STIs and HIV and AIDS. The majority reported that these services were geographically accessible but few were financially accessible to adolescents. However, qualitative data revealed that available services were not specifically provided for adolescents but for general use. Age (*p* = ≤ .05), education (*p* = ≤ .05) and income (*p* = ≤ .05) were found to be significantly associated with access to SRHS.

**Conclusion:**

SRHS were generally physically available but not financially accessible to adolescents. Adolescents’ clinics were not available and this could affect the access of SRHS by adolescents. Education and income were significantly associated with access to SRHS.

## Background

The sexual and reproductive health needs of adolescents are often underserved in many societies [1], yet adolescents constitute large proportion of the population. They represent 25% of the world population [2] and are characterized by series of physiological, psychological and social changes that expose them to unhealthy sexual behaviour such as early sex experimentation, unsafe sex and multiple sexual partners [3]. These put them at high risk of sexual and reproductive health (SRH) problems [1]. Such problems include early marriage, teenage pregnancies, unsafe abortion, sexually transmitted infections (STIs), HIV and AIDS, and other life threatening SRH problems [4].

The high increase in the rate of these SRH problems among young people in sub-Saharan Africa is alarming [5]. This suggests the need for adequate attention towards adolescents’ sexual and reproductive health. Adolescents’ SRH needs and problems are yet to receive adequate attention especially in the developing countries like Nigeria, despite the recognition of youth-friendly reproductive health services as a way of improving their access and utilization of SRH services [6] in order to achieve quality SRH.

Efforts to attain quality sexual and reproductive health are constrained by inadequate access to and inequitable distribution of quality SRH services especially in sub-Sahara African countries. These contribute to poor utilization of SRHS among young people in sub-Saharan African countries [7], resulting to high prevalence of sexual and reproductive health problems especially among the adolescents [8]. An estimate of 333 million new cases of curable STIs occur mostly in developing countries with the highest rate among 20–24 years old, followed by those within the ages of 15 and 19 years [9]. It was also estimated that 1.3 million adolescent girls and 780,000 adolescent boys were living with HIV worldwide, and 79% of new HIV infection among adolescents were in Sub-Saharan Africa [6].

Nigeria has an estimated population of 191, 835, 936 [10] with 22.3% adolescents [11]. One in 20 of these adolescents contracts a sexually transmitted infections each year, and half of all cases of HIV infection take place among people under the age of 25 years [12]. About 40% of new HIV infection occurs among young people in Nigeria [13]. This could result from early sexual debut and early marriage which increase adolescents’ HIV vulnerability. The median age at first sexual intercourse is 17.6 and 21.1 years for women and men respectively, while the median age at first marriage is 18.1 and 27.2 years for women and men respectively [14]. Teenage and unwanted pregnancies are also problems of adolescents especially the unmarried. Although, the abortion law and policy in Nigeria prohibits legal access to legal abortion services, about 1.25 million commit induced abortion yearly by unskilled providers and many have serious complications without obtaining the post abortion care needed [15]. These indicate that the utilization of SRH services by the adolescents in Nigeria is low, arising from disparities in both provision and accessibility of the services and also lack of priority to adolescents’ SRH [16]. Availability and accessibility of quality and affordable SRHS ensure adolescents’ sexual and reproductive health wellbeing [17].

Despite the global promotion of availability of SRH services, most rural areas still lack these services [18]. Moreover, both geographical and financial accessibility to SRH services by the adolescents in low and medium income countries are influenced by different socio-demographic factors [19, 20]. Age and educational status of adolescents were found to affect their use of reproductive health services [21]. This study therefore, assessed the availability and accessibility of SRHS and association between access to SRHS and age, gender, level of education, location and income among adolescents in Enugu State, Nigeria.

## Methods

### Study area and period

This study was conducted in Enugu State, Southeast Nigeria between January 2015 and July 2016. The state comprised 17 Local Government Areas (LGAs) with an estimated total population of 3,267,837 [22]. Of this population, 734,297 (22%) were people of age group 12–22 years; 343,037 (47%) were males and 388,260 (53%) were females. Some of these LGAs have commercial areas like big markets and hotels that attract visitors who come for one business or the other. Adolescents in these areas unlike those in non-commercial areas, engage in a lot of business such as hawking and even commercial sex working. Their males also engage in commercial motor-cycle riding (okada) which exposes them to rough or unhealthy lifestyles. Such activities expose them to unsafe sex and early marriage predisposing them to sexual and reproductive health problems like STIs, and HIV and AIDS, teenage pregnancy, abortion and their consequences. These unhealthy life styles and SRH problems may arise because the young people in this area do not utilize SRH services due to some social and demographic factors. The availability and accessibility of these services to this group of people remained uncertain and therefore, necessitated the present study.

### Design and sampling techniques

A mixed method (quantitative and qualitative) approach was employed. The cross-sectional design was adopted to assess the availability and accessibility of SRH services to adolescents in Enugu State, Nigeria. The sample was 1620 adolescents; 756 (47%) males and 864 (53%) females, and 217 public health facilities. The sample size was determined using Cohen, Manion and Morrison’s sample size chart [23] which suggests that when a population size is five hundred thousand (500,000) and above at (95%) confidence level and (5%) interval level, the sample size should be three hundred and eighty-four (384) and above and when a population size is five hundred (500) and above at (95%) confidence level and (5%) interval level, the sample size should be two hundred and seventeen (217) and above.

Part one: sampling of questionnaire respondents to assess accessibility of SRHS.

Sampling occurred in three stages. First stage sampling involved stratifying the LGAs in each senatorial zone into urban and rural LGAs (2 senatorial zones have six LGAs each, while one senatorial zone has 5 LGAs). The second stage involved selecting one urban and two rural LGAs each from the three senatorial zones using simple random sampling technique of balloting without replacement. This is because each senatorial zone is made up of at least one urban LGA. Two senatorial zones had only one urban LGA each; therefore, the urban LGA in the zone was purposively picked. This sampling gave a total of nine (3 urban and 6 rural) LGAs out of the seventeen LGAs. In each LGA selected, the target sample of adolescents was 84 males and 96 females, which gave a total of 252 males and 288 females from each senatorial zone. The third stage involved selecting six political wards (a geographical area made up of few communities) from each LGA using systematic sampling technique so as to spread the sample selection to a reasonable representation of the LGA. This gave a total of fifty-four (54) wards. The target sample at this stage was 14 males and 16 females from each of the six wards using convenience sampling technique based on accessibility and willingness of the adolescents to participate. This gave the grand total of 1620 (756 males and 864 females) being the sample size used for the study.

Part two: sampling of health facilities to assess availability of SRHS.

In Enugu State, public health facilities are distributed in LGAs by wards. Every ward therefore, has at least one public health facility. Public health facilities (which were used to check the availability of SRHS) were purposively selected for the study since they attract both government and non-governmental support for reproductive health services more than the private facilities. From the 508 public facilities, 217 were selected using proportionate sampling technique thus ensuring that at least one health facility was selected from each ward.

Part three: selection of interview and focus group participants.

Convenience sampling technique was used to select 6–10 male and 6–10 female adolescents from the questionnaire respondents in each of the nine LGAs selected for focus group discussion. This gave 2 groups (1 male group and 1 female group) from each LGA, giving a total of 18 focus groups. Convenience sampling technique was also used to select 3 (1 male and 2 female) interviewees from each LGA for In-Depth Interview (IDI). This gave a total of 27 interviewees.

### Data collection procedure

Checklist, questionnaire, focus group discussion guide and in-depth interview guide were used to collect data from the respondents on both personal and group contacts. The checklist was adapted from WHO’s service availability and readiness assessment core instrument [24]; and was used to collect data and measure availability of SRHS for adolescents in the health facilities. Only the sections that elicit information on availability of the SRHS studied were adapted. The health officers-in-charge of the health facilities sampled were interviewed with the Checklist.

Structured questionnaire was prepared through review of related literature. The questionnaire which contained two parts was used to measure accessibility of SRHS. Accessibility in our study was measured based on proximity of health facilities (can walk to the health facility within 30 min or not; or less than 1 mile) and affordability of the services to adolescents. First part contained the socio-demographic characteristics of the respondents while the second part contained both geographical and financial accessibility of related components of adolescents’ sexual and reproductive health services (sexuality education, family planning services, safe motherhood services, post abortion care and prevention and treatment of STIs and HIV and AIDS). Validity of the instruments was established by five experts. The reliability of the questionnaire was established by pre-testing the questionnaire on 20 adolescents in Anambra State (outside the study area but with the same characteristics with the respondents under study). Kudder-Richardson’s formula 21 (K-R 21) was used to determine the reliability. A reliability coefficient index of .86 was obtained and the instrument was judged reliable for the study. Researchers and nine trained research assistants administered the questionnaire. The research assistants used were below 25 years of age. This was to avoid much age disparity between the research assistants and the respondents in order for the respondents to communicate freely.

Focus Group Discussion (FGD) and In-Depth Interview (IDI) were conducted using already prepared FGD Guide and IDI guide. The FGD and IDI provided detailed information on accessibility of SRHS and addressed issues not covered by the questionnaire. The discussions and interviews were recorded with digital tape recorders. In addition, non-verbal cues from participants were recorded through note taking.

### Data processing and analysis

Data collected were cross-checked for completeness. Logical techniques were employed to identify errors during data cleaning. Out of 1620 copies of questionnaire and 217 checklists used for data collection, only 1447 copies of questionnaire and 192 checklists did not have errors and were used for data analysis. The Statistical Package for the Social Sciences (SPSS) version 20.0 was employed for statistical analysis of quantitative data. Percentages were used to assess the availability and accessibility of SRHS to adolescents, while Chi-square statistic was used to test association between the variables at .05 level of significance. Data from the checklist on availability were presented in Table 2, while data from the questionnaire on accessibility were presented in Tables 3 and 4.

Accessibility in our study was measured both geographically and financially. Adolescents who lived not more than one mile from or could walk to any public health facility where SRHS were provided within 30 min were regarded as having geographical access to SRHS. Affordability (which was reported by the respondents) of travelling cost and costs of services as perceived by the respondents were used to determine the financial accessibility.

The responses from focus group discussion and IDI were transcribed in English language while maintaining the contexts of the responses. The NVivo 11 Pro software was used to code and analyze the data thematically. The data are presented alongside the quantitative findings.

## Results

Table 1 shows the socio-demographic data of the study sample that responded to the questionnaire. Among participants (*n* = 1447), males constituted 42.9% while females constituted 57.1%. Their age ranged from 12 to 22 years with a mean age of 16.9 years. Most of the participants had secondary education (54.0%) and most were Christians (96.3%). Greater proportions of the participants were living with their parents (62.3%) and were single (86.8%). Majority had a monthly income less than ₦5000.00 (1 USD = 199.3 NGN).

**Table 1:** Socio-Demographic Characteristics of Adolescents that Responded to the Questionnaire on Accessibility of SRHS (*n* = 1447)

S/N	Characteristics		**%**
1	Gender		
	Male		42.9
	Female		57.1
	Total		**100.0**
2	Age		
	12–16		48.2
	17–22		51.8
	Total		**100.0**
3	Education		
	Primary		2.2
	Secondary		54.0
	Tertiary		42.0
	None		1.8
	Total		**100.0**
4	Religion		
	Christianity		96.3
	Islam		1.5
	African Traditional Religion		2.1
	Total		**100.0**
5	Location		
	Urban		43.3
	Rural		56.7
	Total		**100.0**
6	Living Status		
	With parents		62.3
	Alone		20.4
	With friends/husband		3.2
	In school		14.1
	Total		**100.0**
7	Marital Status		
	Married		12.4
	Single		86.8
	Divorced		.4
	Separated		.3
	Total		**100.0**
8	Parity (females only)		
	None		81.5
	1–3		13.8
	4–6		3.0
	7 and above		1.7
	Total		**100.0**
9	Monthly Income		
	Below ₦1000.00 k		46.8
	₦1000.00 k-₦4000.00 k		19.8
	₦5000.00 k-₦10,000.00 k		16.0
	₦11,000.00 k-₦20,000.00 k		8.8
	Above ₦20,000.00 k		8.6
	Total		**100.0**

Note: 1 USD = 199.3 NGN; for parity, *N* = 826

Table 2 presents data from the health facilities on availability of SRHS. The table shows that 55.8% of the health facilities had sexuality education services, 57.1% had family planning information and services, 86.7% had safe motherhood services, and 67.5% had services for prevention and management of STIs and HIV and AIDS.

**Table 2:** Percentage of sampled facilities that report availability of SRHS in Enugu State (*n* = 192)

S/N	Items				
			**%**		
	Sexuality Education Services				
1	Trained sexuality education provider		60.9		
2	Education on human biology		51.0		
3	Education on puberty and menstrual hygiene practices for youth		73.4		
4	Education on skills to overcome sexual desires for youth		42.7		
5	Education on healthy Associations for youth		42.7		
6	Education on dangers of premarital and unsafe sex for youth		72.9		
7	Information on reproductive rights and policy for youth		34.4		
8	Information on harmful cultural practices like female circumcision		65.6		
9	Information on prevention of non-infectious conditions of reproductive health such as fistula and cancers		58.9		
	Cluster % Total		**55.8**		
	Family Planning Information and Services				
10	Trained family planning provider		59.9		
11	Family planning information		80.7		
12	Oral pills		61.5		
13	Injectable contraceptives		52.6		
14	Male condoms		81.8		
15	Female condoms		70.8		
16	Intrauterine contraceptive device (IUCD)		27.1		
17	Emergency contraceptives		22.4		
	Cluster % Total		**57.1**		
	Safe Motherhood Services				
18	Trained midwife		62.5		
19	Antenatal services for pregnant youth		92.7		
20	Safe delivery services for youth		92.7		
21	Postnatal services for youth		87.5		
22	Immunization services		92.7		
23	Growth monitoring services		93.2		
24	Information on infant feeding practices		85.9		
	Cluster % Total		**86.7**		
	Post Abortion Care (PAC) Services				
25	Trained PAC provider		19.3		
26	Emergency health care, in cases of bleeding and shock		69.8		
27	Manual vacuum aspiration (evacuation) of retained product of conception		30.7		
28	Information on prevention and management of STIs and HIV and AIDS for youth		71.4		
	Cluster % Total		**47.8**		
	Prevention and Management of STIs and HIV and AIDS Services				
28	Trained HIV and AIDS services provider		63.0		
29	Information on prevention and management of STIs, HIV and AIDS for youth		89.6		
30	Voluntary counseling and testing for youth		83.3		
31	Antiretroviral therapy for youth		35.4		
32	Services for the prevention of mother-to-child transmission of HIV and other STIs		64.1		
33	Condoms for sexually active youth		69.3		
	Cluster % Total		**67.5**		

Tables 3 and 4 present data on accessibility of SRHS. Table 3 shows that overall geographical and financial accessibility of SRHS was 58.4 and 50.5% respectively. More participants viewed sexuality education to be geographically (66.7%) and financially (58.7%) accessible. Family planning was viewed to be only geographically accessible (51.9%). Safe motherhood services were considered by majority to be accessible geographically (70.6%) and financially (61.7%). Post abortion care services were viewed by 51.0% to be geographically accessible and prevention and management of STIs and HIV and AIDS services were considered geographical accessible by 51.6% of the respondents.

**Table 3:** Percentage of Sampled Adolescents that Reported Accessibility of SRHS in Enugu State (*n* = 1447)

S/N	Items	Geographical Access %				Financial Access %			
	Sexuality Education Services such as education on								
1	human biology		70.0				60.8		
2	Puberty		72.3				62.4		
3	menstrual hygiene		70.4				63.2		
4	skills to overcome sexual desires		61.2				56.4		
5	healthy Associations		61.2				52.8		
6	dangers of premarital and unsafe sex		65.2				56.5		
	Cluster % Total		**66.7**				**58.7**		
	Family Planning Information and Services such as								
7	Condoms		72.8				65.9		
8	Oral pills		52.6				44.9		
9	Injectable contraceptives		45.0				35.0		
10	Intrauterine contraceptive device (IUCD)		37.2				29.2		
	Cluster % Total		**51.9**				**43.8**		
	Safe Motherhood Services such as								
11	Antenatal		75.5				63.9		
12	Safe delivery		69.8				60.5		
13	Postnatal		66.3				57.1		
14	Immunization		77.2				69.5		
15	Infant feeding information		64.2				57.7		
	Cluster % Total		**70.6**				**61.7**		
	Post Abortion Care (PAC) Services such as								
16	Emergency care during bleeding		50.5				41.2		
17	Manual removal of retained product of conception		35.9				30.2		
18	Information on the prevention of unwanted pregnancy		59.0				52.1		
19	Information on prevention of abortion		58.7				51.8		
	Cluster % Total		**51.0**				**43.8**		
	Prevention and Management of STIs and HIV and AIDS Services such as								
20	Voluntary counseling and testing		67.0				56.3		
21	Provision of antiretroviral therapy (ART)		36.0				32.9		
22	Treatment of STIs		49.1				41.1		
23	Supply of condoms		54.0				47.3		
24	prevention of mother-to-child transmission of HIV and other STIs		52.0				45.5		
	Cluster % Total Overall % Total		**51.6** **58.4**				**44.6** **50.5**

**Table 4:** Association between Socio-Demographic Factors and Access to SRHS among Adolescents in Enugu State (n = 1447). Socio-Demographical Factors Associated with SRHS

Factors	Sexuality Education						Family Planning						Safe Motherhood						Post Abortion Care						Prevention and Management of STIs, HIV and AIDS					
	Geographical Access			Financial Access			Geographical Access			Financial Access			Geographical Access			Financial Access			Geographical Access			Financial Access			Geographical Access			Financial Access		
	%	*χ* ^2^	*P*	%	*χ* ^2^	*p*	%	*χ* ^2^	*P*	%	*χ* ^2^	*P*	%	*χ* ^2^	*p*	%	*χ* ^2^	*p*	%	*χ* ^2^	*P*	%	*χ* ^2^	*P*	%	*χ* ^2^	*P*	%	*χ* ^2^	*p*
	AC			AF			AC			AF			AC			AF			AC			AF			AC			AF		
Gender																														
Male	72.6	7.477	.006*	69.6	.921	.337**	61.7	.587	.443**	53.9	5.748	.017*	71.8	1.144	.285**	62.0	.176	.675**	63.4	.145	.704**	57.5	.831	.362**	55.1	2.122	.145**	50.9	11.561	.001*
Female	78.8			67.2			59.7			47.6			74.3			63.1			62.9			55.1			51.2			41.9		
Age																														
12–16	72.9	7.937	.005*	65.6	4.333	.037*	52.7	34.996	.000*	41.6	40.753	.000*	66.1	34.741	.000*	55.8	26.576	.000*	59.8	5.398	.020*	51.1	13.871	.000*	51.2	1.467	.226**	42.0	7.468	.006*
17–22	78.8			70.7			67.9			58.4			79.9			68.9			65.7			60.8			54.4			49.2		
Level of education																														
Primary	53.1	26.417	.000*	46.9	22.245	.000*	56.2	45.767	.000*	37.5	47.201	.000*	65.6	29.136	.000*	50.0	26.711	.000*	46.9	16.076	.001*	43.8	26.103	.000*	37.5	4.405	.221**	34.4	4.764	.190**
Secondary	73.0			64.8			53.1			43.0			68.5			57.4			59.8			51.0			52.0			44.3		
Tertiary	81.9			74.2			70.7			60.9			80.4			70.2			68.3			63.8			54.9			48.5		
None	65.4			57.7			50.0			38.5			57.7			57.7			50.0			46.2			50.0			38.5		
Location																														
Urban	77.4	.870	.351**	64.0	9.237	.002*	61.9	.835	.361**	50.2	.002	.962**	71.6	1.527	.217**	54.4	31.985	.000*	65.7	3.773	.052**	55.3	.269	.604**	58.1	11.943	.001*	44.5	.699	.403**
Rural	75.2			71.5			59.5			50.4			74.5			68.9			60.7			56.7			48.9			46.7		
Income(₦)																														
<1000	72.4	11.193	.024*	65.7	9.765	.045*	54.1	42.677	.000*	45.2	32.052	.000*	65.6	38.675	.000*	57.0	19.113	.001*	57.3	19.573	.001*	53.2	10.363	.035*	54.8	7.277	.122**	47.9	10.349	.035*
1000–4000	78.7			71.7			59.1			47.6			80.4			70.3			66.4			57.0			49.7			44.4		
5000–10,000	79.7			66.8			62.9			51.3			78.4			67.7			65.5			54.7			48.3			38.4		
11,000–20,000	78.1			78.1			78.1			65.6			80.5			64.8			73.4			67.2			50.8			43.0		
>200,000	76.6			66.1			76.6			66.1			81.5			63.7			69.4			61.3			60.5			54.0		

*AC* Accessible, *AF* Affordable, *χ*^2^ = Chi-square, p = *p*-value, *significant, **Not significant, at .05 level of significance, 1USD = 199.3NGN

*AC* Accessible, *AF* Affordable, *χ*^2^ Chi-square, *p* p-value, *significant, **Not significant at .05 level of significance

Table 4 shows that there is significant association between both geographical and financial access to sexuality education, family planning, and safe motherhood services and age (*p* ≤ .05), level of education (*p* ≤ .05), and income (*p* ≤ .05). Older adolescents (17–22 years) had more access to the services than the younger adolescents. There is also significant association between both geographical and financial access to post-abortion care and age (*p* ≤ .05), level of education (*p* ≤ .05) and income (*p* ≤ .05). There are variations in the levels of education and income of the respondents and their access to the services.

### Qualitative data

#### Availability of SRHS for adolescents

In-depth interview reveals that adolescents interviewed agreed that some of the SRHS were available but not particularly for adolescents. The available services for adolescents reported were: sexuality education which is provided in the secondary schools through other health-related subjects, and services for prevention and management of STIs and HIV and AIDS, which are mainly provided by churches and schools during youth week. In the words of some interviewees:

“*I get sexuality education services and services for prevention and management of STIs and HIV and AIDS in the school and church during youth’s week but for others, I don’t know about them”* (Udenu 002). On the issue of family planning and safe motherhood services, participants were of the view that the services were only available for married women. One of the interviewee said *“I have not received such (family planning services), and I don’t think it is made for adolescents. It is only for married couples” (Enugu-North 003)*. Another interviewee said *“safe motherhood services are for married mothers not for us but I know that the services are available in the health centers for all pregnant women”* (Isi-Uzo 001). Interviewees reported non-availability of post-abortion care. An interviewee said *“I have not heard of post abortion care services and I know there is nothing like that in the health centers”* (Igbo-Eze South 001)*.*

Similarly, focus group discussion revealed that participants in the 18 groups (male and female) reported that the available SRHS for adolescents are sexuality education and services for prevention and management of STIs and HIV and AIDS which they receive in schools and churches. One participant said *“Yes sexuality education services and screening services for STIs and HIV/AIDS are being provided for us in school and church during youth week”* (Ezeagu Male FGD-P1). The participants had not heard of post-abortion care (PAC). A participant said *“Am hearing the PAC services for the first time”*(Nsukka Female-FGD-P4) and another male participant from FGD said *“No o! There is nowhere these services are provided for the adolescents. I have not seen”* (Nsukka Male FGD-P1).

#### Accessibility of SRHS to adolescents

In-depth interview show that interviewees have geographical access to public health facilities that provide general SHRS, as they indicated that they can walk to the health facility within 30 min. One interviewee said, *“Yes for me I can trek within 30 minutes because I stay near the hospital” (Enugu-North 001)*. However, participants revealed that not all the SRHS were available in the health facilities and the accessible SRHS were not for adolescents alone. On financial accessibility, few interviewees indicated that they could afford the cost of the SRHS. An interviewee said *“…these services are not fully accessible to me because I can’t afford the cost of the services”(Isi-Uzo 003).*

FGD participants indicated that available SRHS were geographically accessible except in rural areas where some accessible health facilities do not provide some of the SRHS but no affordable. In their words, *“Some are cheap while some are not like services for prevention and management of STIs and HIV and AIDS”* (Nsukka Male FGD-P5*). “……but the ones I have accessed are not cheap to me, may be because I am a student”* (Isi-Uzo Female FGD-P1).

## Discussion

For adolescents to use SRHS and lead a healthy sexual and reproductive live, SRHS have to be available and adequately accessible. Addressing the problem of availability and accessibility of SRHS for adolescents is essential to increasing adolescents’ utilization of SRHS. Most peer reviewed literature used quantitative data only. Quantitative data only seem not to get adequate SRH information from adolescents especially in developing countries like Nigeria, where culture and tradition still affect adolescents’ SRH. This study was therefore, aimed at assessing SRHS available and accessible to adolescents in Enugu State, using mixed method (quantitative and qualitative).

The secrecy accorded to sexual issues in the study area was a challenge to this study and might be the reason for some differences in qualitative and quantitative data. The study was delimited to adolescents in Enugu State, and therefore, may not be used to generalize to all adolescents in a multi-ethnic country like Nigeria.

The available SRHS were sexuality education, family planning, safe motherhood, and prevention and management of STIs, and HIV and AIDS, most of which are provided in schools and churches. Previous studies also reported schools and churches as most important community sources of sex education [25, 26]. Moreover, family planning and safe motherhood services were provided in almost all the primary health centers in the state. However, these services were general services and not specifically for adolescents as revealed by qualitative data and absence of youth clinic or unit in all the health facilities visited. The finding was at variance with previous studies that reported lack of SRHS [27, 28]. Qualitative data revealed that the most available SRHS for adolescents were sexuality education services and services for prevention and management of STIs, HIV and AIDS provided in schools and churches during youth weeks and not in the health facilities [29]. This finding could be due to the fact that adolescent/youth clinics or units were lacking in the political wards and health facilities visited. The qualitative data were therefore, in support of the findings of similar studies which stated that only few health facilities provided the essential SRH services for young people [7, 27].

All the available SRHS were geographically accessible while only two (sexuality education and safe motherhood services) were financially accessible. Every political ward we visited had at least one primary health facility. Sexuality education was financially accessible because it was provided mainly in the churches and schools and no further payments apart from school fees were required. Safe motherhood services were also financially accessible because government provided these services at a much reduced price and sometimes free. The finding was consistent with the assertion that young people should have universal access to SRHS [30, 31]. The finding was, however, at variance with the finding of low accessibility of SRHS to adolescents reported by previous studies that utilized quantitative data [18, 32].

Our qualitative findings from IDI revealed that more than half (55.6%) of the interviewees had geographical access to SHRS as they indicated that they could walk to the health facility within 30 min. However, most of the interviewees revealed that not all the SRHS were available in the health facilities [33] and some of the accessible SRHS were not for adolescents alone, but rather for everybody. On financial accessibility, slightly more than one third (37.0%) of the interviewees indicated that they could afford the cost of the SRHS. Data collected through FGD revealed that majority of both male and female participants indicated that available SRHS were geographically accessible except in rural areas where some accessible health facilities do not provide some of the SRHS. This finding opposes previous assertion that costs of services were barriers to adolescents’ access to SRHS [34]. Though, these services according to the participants are affordable, they are provided free to only pregnant women.

Statistically significant associations exist between both geographical and financial access to sexuality education, family planning, safe motherhood services and post-abortion care and age, level of education and income. This implies that age, education and income can influence adolescents’ access to sexuality education, family planning, safe motherhood and post abortion care. Older adolescents accessed the services more than the younger ones. Older adolescents are more independent than the younger ones and so could decide to access or not to access SRHS. The finding is consistent with previous assertion that level of access of health services is lower in younger adolescents [35]. It was also observed during in-depth interview and focus group discussion that younger participants were silent and of little knowledge of majority of issues discussed [36]. The finding could also be related to poor sexuality education at home and even in schools. Some parents may not give their children age appropriate sex education which should begin at home. It has also been observed by that some schools do not teach sex education as a separate subject but subsumed under other subjects.

Our study found that respondents with secondary education accessed SRHS more than those with other levels of education, even tertiary. This was at variance with the assertion that more educated people are more likely to access health care and understand with self-confidence to act on them [37]. Similar study opined that education can lead to higher ability to process health-related information and also influence an individual’s preference for future, which in turn improves his or her health behaviour and health outcomes [38–40].

Furthermore, the significant association between income and access (financial) to SRHS in this study is uncommon. Respondents with income between ₦5000 and ₦10,000 had more financial access to SRHS than those with income above ₦10,000. Previous studies show higher access with higher income levels [41, 42]. However, lower income negatively influenced adolescents’ access to the services [43]. Our qualitative data revealed that sexuality education and prevention and management of STIs and HIV and AIDS were provided mostly in schools and churches for both male and female adolescents. However, some male participants in the FGD were of the opinion that SRH services were meant for girls only. Boys perceived SRHS as designated for girls [44]. This finding is however, similar to the findings of studies conducted in Nigeria [45] and Ethiopia [21] that reported association between gender and access and utilization of health services. The finding is not consistent with a study that reported no difference between gender and access to SRHS [18].

## Conclusions

We found that the majority of the SRH services were available and geographically accessible, but very few were financially accessible to adolescents. These services were not specifically for the adolescents and therefore, may hinder their access as well as utilization. Socio-demographic factors associated with adolescents’ access (geographical and financial) to SRH services were age, education and income. We therefore, suggest that adolescents-friendly SRH services should be made available and accessible.

## Abbreviations

FGD: Focus Group Discussion
HIV and AIDS: Human Immunodeficiency Virus and Acquired Immune Deficiency Syndrome
IDI: In-depth Interview
LGA: Local Government Area
SPSS: Statistical Package for the Social Sciences
SRH: Sexual and Reproductive Health
SRHS: Sexual and Reproductive Health Services
STIs: Sexually Transmitted Infections
